# Sequencing our way to more accurate community abundance

**DOI:** 10.1111/1755-0998.13717

**Published:** 2022-10-22

**Authors:** Georgina L. Brennan

**Affiliations:** ^1^ Institute of Marine Sciences (ICM), CSIC Barcelona Spain

**Keywords:** Community Ecology, Metagenomics, Metabarcoding, phytoplankton

## Abstract

Over the last two decades, there has been a huge increase in our understanding of microbial diversity, structure and composition enabled by high‐throughput sequencing technologies. Yet, it is unclear how the number of sequences translates to the number of cells or species within the community. In some cases, additional observational data may be required to ensure relative abundance patterns from sequence reads are biologically meaningful. The goal of DNA‐based methods for biodiversity assessments is to obtain robust community abundance data, simultaneously, from environmental samples. In this issue of *Molecular Ecology Resources*, Pierella Karlusich et al. (2022) describe a new method for quantifying phytoplankton cell abundance. Using *Tara* Oceans data sets, the authors propose the photosynthetic gene *psbO* for reporting accurate relative abundance of the entire phytoplankton community from metagenomic data. The authors demonstrate higher correlations with traditional optical methods (including microscopy and flow cytometry), using their new method, improving upon molecular abundance assessments using multicopy marker genes. Furthermore, to facilitate application of their approach, the authors curated a *psbO* gene database for accessible taxonomic queries. This is an important step towards improving species abundance estimates from molecular data and eventually reporting of absolute species abundance, enhancing our understanding of community dynamics.

High‐throughput sequencing technologies for identification of taxa from environmental samples have significantly improved our understanding of biodiversity and community assembly processes. However, quantification of species abundance from sequence reads is not a straightforward task. This is because biases from DNA extraction, PCR amplification and sequencing will affect the number of sequence reads obtained for each taxonomic unit and therefore the representation within the environmental sample (Bik et al., 2012). In addition, multicopy markers are often targeted to increase detection sensitivity of target DNA from environmental samples, for example prokaryote (16S) and eukaryote (18S) rRNA marker genes. However, reporting the abundance of sequences from multicopy molecular markers may not be an accurate measure of the abundance of organisms containing those sequences and this is because of large variations in copy number within and between taxa (Figure 1).

**FIGURE 1:**
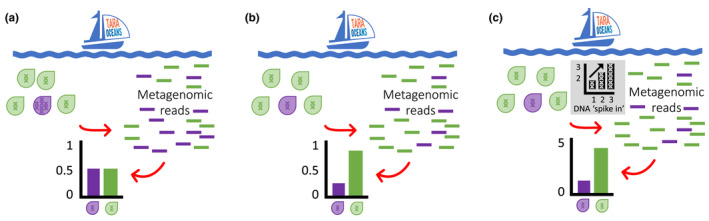
Abundance of two marine microbes illustrated by three hypothetical scenarios: (a) equal relative abundance does not match cell abundance due to copy number variation of the target molecular marker (e.g., rRNA marker genes), (b) accurate relative abundance matches cell abundance when using single‐copy molecular markers (e.g., *psb0* gene) and (c) accurate absolute abundance when using single‐copy molecular markers and a DNA spike‐in.

Inaccurate assessments of abundance will have serious consequences for our understanding and management of ecosystems. For example, Pierella Karlusich et al. (2022) highlight the ecological importance of marine phytoplankton, including their position at the foundation of ocean ecosystems and their roles in primary productivity and biogeochemical cycles (Field et al., 1998). Under future global change species sorting will potentially alter the composition of functional groups within marine microbial communities (Di Pane et al., 2022), which in turn feeds back into the biogeochemical cycles. It is therefore important to know how these communities will be composed in the future, and the consequences for the ecosystem services they provide. Targeted amplicon sequencing (“metabarcoding”) is now routinely used for the characterization of complex assemblages of prokaryotic and eukaryotic organisms (Creer et al., 2016) and we are now in a position where we can reliably identify most of the abundant taxa in complex assemblages (albeit with some exceptions) and provide “semiquantitative” data of taxon abundance from complex mixtures, for example the ocean microbiome (Giner et al., 2016), soil microbiome (Delgado‐Baquerizo et al., 2018) and air microbiome (Drautz‐Moses et al., 2022). However, it is well documented that metabarcoding suffers from biases associated with PCR amplification of target genes (Bik et al., 2012). Metagenomic sequencing (the sequencing of genomic fragments from many members of the community) is a nontargeted, PCR‐free method and, as costs decline, is an emerging solution to taxonomic identification without biases introduced by PCR. Whilst traditional methods, such as microscopy and flow cytometry, are better at providing quantitative data and are well validated, they often lack the ability to scale up to whole communities, especially in systems or with methods that rely on human expertise instead of automation (Makiola et al., 2020). The goal is to obtain reliable abundance data for each taxonomic unit, from the number of sequences reads obtained from the environmental sample.

One method to robustly measure relative abundance from environmental samples has been proposed by Pierella Karlusich et al. (2022) where they describe each step of their selection and validation process. Using data sets from the *Tara* Oceans—global expedition sampling global plankton in the upper layers of the world ocean (Sunagawa et al., 2020; Pierella Karlusich et al. 2022) target nuclear‐encoded single‐copy, core, photosynthetic genes obtained from metagenomes to circumvent the limitations of targeted gene sequencing (metabarcoding) and multicopy markers. The authors focused on the *psbO* gene, which is essential for photosynthetic activity and does not have nonphotosynthetic homologues, so can be used to measure abundance of the total photosynthetic group and has the added benefit of covering the whole phytoplankton community. Similarly, both cyanobacteria and eukaryotic phytoplankton can be measured by combining two rRNA marker genes (e.g., prokaryotic 16S and eukaryotic 18S), although relative abundances derived from different amplicon libraries cannot be directly compared (Tkacz et al., 2018). Importantly, cross‐domain comparisons can be made using the *psbO* gene.

Using the *psbO* gene, Pierella Karlusich et al. (2022) were able to examine the biogeography of the entire phytoplankton community simultaneously. To validate their approach, the authors used *Tara* Oceans data, including imaging data sets (microscopy and flow cytometry) and molecular data sets from metabarcoding, metagenomics and metatranscriptomics. Using imaging data sets (flow cytometry, microscopy) they demonstrated the accuracy of their approach and even confirmed the presence of colony formation and symbiosis in some of the smallest phytoplankton cells that were found in the largest size‐fractioned water samples. Armed with the evidence to demonstrate that the *psbO* gene accurately provides relative abundance data, the authors compared their results with the commonly used rRNA marker genes 16S and 18S (rRNA gene miTags from metagenome data and rRNA gene metabarcoding). Here they show that the *psbO* gene outperformed rRNA gene data sets in reporting accurate relative abundance of phytoplankton. Furthermore, the authors demonstrate that the *psbO* gene improves measures of microbial community diversity, structure and composition as compared to rRNA genes, and identified biases in metabarcoding data sets. However, they report that diversity indices such as Shannon diversity (which accounts for both species richness and evenness) were sufficiently robust to account for biases introduced by the rRNA marker methods. Furthermore, they confirm that neither rRNA gene markers nor *psbO* could accurately report biovolume.

This is an exciting tool since we still do not have a clear understanding of the abundance of phytoplankton groups from the ocean. Similarly, the same steps can be followed from Pierella Karlusich et al. (2022), in order to identify suitable genes for other study systems. There are many research avenues where the use of good‐quality abundance data would be enormously impactful: for example, to make more accurate assessment of floral resource use from pollen grains found in honey (Jones et al., 2021) or the bodies of pollinators (Lowe et al., 2022), exploring how the abundance of allergenic airborne pollen correlates with human health (Rowney et al., 2021), and to gain insights into the relationship between gut microbiome and human health (Proctor et al., 2019). However, it is important that new markers are accompanied by well‐populated genetic databases in order to avoid biases during taxonomic assignment. Measuring absolute abundance is the ultimate goal since variations in relative abundance may not reflect absolute abundance and can skew our understanding of community structure and dynamics (e.g., when one taxonomic unit increases in relative abundance, another necessarily decreases). Future investigations using this approach can achieve absolute abundance using careful sampling design and DNA internal standards (“spike‐in”) (Tkacz et al., 2018) (Figure 1).

## CONFLICT OF INTEREST

The author declares no conflicts of interest.
